# A behavioural syndrome, but less evidence for a relationship with cognitive traits in a spatial orientation context

**DOI:** 10.1186/s12983-017-0204-2

**Published:** 2017-03-24

**Authors:** Andrea C. Schuster, Uwe Zimmermann, Carina Hauer, Katharina Foerster

**Affiliations:** Department of Comparative Zoology, Institute for Evolution and Ecology, University of Tübingen, Auf der Morgenstelle 28, D-72076 Tübingen, Germany

**Keywords:** Animal personality, Spatial learning, Cognitive styles, Speed-accuracy trade-off, Eurasian harvest mouse, *Micromys minutus*

## Abstract

**Background:**

Animals show consistent individual behavioural differences in many species. Further, behavioural traits (personality traits) form behavioural syndromes, characterised by correlations between different behaviours. Mechanisms maintaining these correlations could be constrained due to underlying relationships with cognitive traits. There is growing evidence for the non-independence of animal personality and general cognitive abilities in animals, but so far, studies on the direction of the relationship between them revealed contradictory results. Still, it is hypothesised that individuals may exhibit consistent learning and decision styles. Fast behavioural types (consistently bolder and more active individuals) are expected to show faster learning styles. Slow behavioural types in contrast are assumed to learn slower but more accurately. This can be caused by a speed-accuracy trade-off that individuals face during decision making. We measured the repeatability of three personality and four spatial cognitive traits in adult Eurasian harvest mice (*Micromys minutus*). We analysed correlations among personality traits (behavioural syndrome). We further investigated the relationships between personality and spatial cognitive traits as a first step exploring the potential connection between personality and cognition in this species.

**Results:**

Our results showed that exploration, activity and boldness were repeatable in adult mice. Spatial recognition measured in a Y Maze was also significantly repeatable, as well as spatial learning performance and decision speed. We found no repeatability of decision accuracy. Harvest mice showed a behavioural syndrome as we observed strong positive correlations between personality traits. The speed-accuracy trade-off was not apparent within, nor between individuals. Nevertheless, we found weak evidence for a relationship between personality and spatial cognitive traits as fast behavioural types learned a spatial orientation task faster than slow types, and shyer harvest mice made decisions quicker than bolder mice.

**Conclusions:**

Given these correlations, our data provided some first insights into the relationship between personality and spatial cognitive traits in harvest mice and will hopefully stimulate more studies in this field.

**Electronic supplementary material:**

The online version of this article (doi:10.1186/s12983-017-0204-2) contains supplementary material, which is available to authorized users.

## Background

Consistent individual differences in behaviour are known as animal personality [47, 62]. Further, correlations between consistent behavioural traits – also called personality traits – are defined as behavioural syndrome [52]. In birds, the relation between different personality traits followed a fast-slow continuum [56]. For instance, more exploratory great tits (*Parus major*) were bolder [59], more aggressive [10] and showed more risk-taking behaviours [58] than less explorative birds. In rodents, Koolhaas et al. [36] termed a similar behavioural syndrome the proactive-reactive syndrome, where proactive individuals were more active and more aggressive than reactive mice (*Mus musculus*). The behaviour pattern that an individual expresses was defined as a behavioural type, representing the characteristics of an individual’s personality [3]. Selection for proactive (fast) behavioural types is likely in stable environments, where risky behaviours confer advantages in competitive situations. Reactive (slow) behavioural types, on the other hand, would have advantages in unstable environments which favour behavioural flexibility [36].

The concept of behavioural types can also be applied to cognitive behaviour [53]. If adaptive cognitive behaviour can be achieved through different strategies, or if animals face consistent trade-offs when solving cognitive tasks, we would expect to observe cognitive styles. Cognitive traits in general provide the basis of any other behaviour as they refer to the capacity of individuals to acquire, process, store and remember information [49]. Cognitive styles refer to *how* individuals acquire, process, store, and remember information, and these strategies are expected to be consistent across time and contexts [53]. Despite the clear theoretical expectation, the existence of cognitive styles in various species still remains to be shown. Consistent cognitive styles can arise, for instance, due to constant decision-making behaviour. When an individual has to take a decision it faces the fundamental problem of a speed-accuracy trade-off [11]. Individuals can either decide fast at the potential cost of an accurate decision or take their time and decide more accurately [8]. Accuracy has been interpreted in the past as a limit to the cognitive ability of an individual [11], making strong selection for accuracy unlikely. However, recent data showed that both cognitive styles (fast vs. accurate) can occur in a population side by side [60], indicating that both styles might be similar adaptive under specific environmental conditions.

Sih and Del Giudice [53] provided a clear theoretical framework for a link between behavioural types and cognitive styles based on a risk-reward trade-off. Fast behavioural types (consistently bolder, more aggressive and more active individuals) are expected to take higher risks while being rewarded faster (fast learning styles). Slow behavioural types in contrast are assumed to decide more accurately but less fast, and to take less risks [53]. Recent tests on correlations between personality types and cognitive traits showed contradictory results: Experimental work on black-capped chickadees (*Poecile atricapillus*) showed that slow exploring individuals showed higher accuracy levels than fast exploring birds when learning an instrumental discrimination task [31]. Bousquet et al. [6] on the contrary could not identify any correlation between exploration and decision accuracy in a spatial learning task in mallards (*Anas platyrhynchos*). Further, Mamuneas et al. [39] did not find any differences in decision making accuracy between shy and bold three-spined sticklebacks (*Gasterosteus aculeatus*). Alternatively, since proactive (fast) individuals are more active and explore novel environments faster, they might also be able to learn spatial tasks faster. For instance, Guenther et al. [28] showed that boldness, activity and aggressiveness correlated positively with association learning ability in wild cavies (*Cavia aperea*). In starlings (*Sturnus vulgaris*), fast explorers were also faster in learning to obtain a food reward [5]. Under a speed-accuracy trade-off, fast learning individuals can be expected to make more errors when recalling the learned information. However, bold and fast learning guppy females (*Poecilia reticulate*) decided more accurately in an association task, where fish learnt to associate a feeding chamber with a more profitable foraging patch [57]. There is a growing interest in the individuality of cognitive abilities within animal species and indications for a relationship between personality and general cognitive ability accumulate, however, it seems hard to predict the direction of these relationships in specific species, and if those are even causal [27].

Like behavioural types, cognitive styles are assumed to show temporal repeatability and cross-context consistency [53]. The rank order of individuals is assumed to be stable between situations, even if the mean behavioural trait expression might differ between tests [52]. These properties have been studied thoroughly for personality traits [4, 7], but so far, only few tests for repeatability and consistency of cognitive styles and of cognitive traits have been published [20, 27, 31, 60].

Spatial orientation skills can be essential for a maximal individual fitness gain and are assumed to be favoured differently depending on each species’ ecology [50]. Spatial learning refers to processes through which animals can encode information about the environment, can navigate through their habitat, and can recall locations of important stimuli within space [23]. Like any other learning process it can be influenced by constant individual behaviour, as new situations are differently encountered, perceived, and assessed by different behavioural types, which can result in different learning outcomes [53]. Furthermore, any learning event itself is part of the individual experience, which further regulates individuality of behaviour [55]. Therefore, spatial learning is one of the suitable candidates for studying the relationship between personality and cognition.

Here, we investigated spatial cognitive traits in a laboratory population of the Eurasian harvest mouse (*Micromys minutus*; Pallas, 1771). We add to the scarce knowledge about repeatability of cognitive traits in animals focusing on spatial orientation abilities. The Eurasian harvest mouse lives in high grass vegetation of wetland areas, but may also occur in grain fields and ruderal areas ([21, 54, 22]). We assume that this species is exposed to a high selection pressure on spatial orientation abilities due to its complex three-dimensional use of habitats. The hippocampus of mammals is involved in the processing of spatial information about the environment [63]. Interestingly, the hippocampus of harvest mice occupied 16.2% of the telencephalon, which is, e.g., 4.6% more than in laboratory mice (*Mus musculus*; 11.6% hippocampal volume of the telencephalon; [61]). This may indicate that the brain structure of harvest mice is specifically adapted to spatial orientation. We investigated individual differences in spatial recognition, spatial learning performance, decision speed, and decision accuracy. We further evaluated the evidence for a behavioural syndrome in this species, based on the previously described personality traits boldness, activity, and exploration [48]. We finally investigated the repeatability of the measured cognitive traits, as well as their relationship with personality traits.

As variable environments may favour different behavioural types and cognitive styles in populations, it is likely that these also occur in harvest mice, as this species inhabits unstable environments [44]. We expected that the three measured personality traits boldness, activity and exploration correlate positively and form a fast-slow-behavioural syndrome [56]. We assumed that mice with better spatial recognition would learn an orientation task faster due to improved spatial cognitive abilities. We further predicted that spatial recognition and spatial learning performance correlate with personality traits. As our cognitive test design was based on active exploration, we assumed a positive relationship between active personality types and spatial learning performance [53]. Finally, we expected a speed-accuracy trade-off as suggested by Sih and Del Giudice [53]. We hypothesised that bolder, more active and more explorative individuals decide faster but less accurately in a spatial learning task. We also hypothesised that fast learners compromise on adequately memorizing information and show less decision accuracy.

## Methods

### Study species

The Eurasian harvest mouse is one of the smallest rodents in Europe with an average body mass of 7.3 g (own measurements of 96 adult individuals, range: 5.2 g to 11.4 g). It inhabits reed and sedge zones, where it nests mainly within a metre above the ground [54, 22]. Its morphological adaptations to this ecological niche are the small body size and the long, prehensile tail for climbing [24]. In the wild, the life span of harvest mice is assumed to range between two and 18 months due to the stochastic nature of the potentially very high predation risk, and to harsh environmental factors [37, 44].

### Housing conditions

The harvest mice tested in this study stemmed from our laboratory population whose founding individuals (*N* = 26) originated from four different zoo populations. All individuals were born in Tübingen between 2011 and 2014. Mice were housed in polycarbonate cages of 60 cm length, 40 cm width and 58 cm height. Individuals were either kept separately or in pairs of equal sex with water and food (hay, grain seeds, fresh fruit and vegetables) ad libitum. The back wall of the cage was covered by a coco coir mat for climbing. For environmental enrichment, cages were additionally equipped with an artificial nest, a running wheel, a paper tube, a wooden branch and a sheaf of wheat, oat and spelt. All mice were kept at constant temperature (22 °C, range: 21.0 °C to 23.5 °C) and light-dark cycle (LD 12:12 h). Animal husbandry and behavioural tests (see below) were permitted by the Regierungspräsidium Tübingen – Referat 35, reference number ZO 2/11.

### Behavioural tests

Standard behavioural tests, originally established to test emotionality in laboratory strains of mice and rats, were modified and adapted to our study species. It was not possible to record data blind because our study involved focal animal observations, however, we included the identity of the observer (one to five different persons per test) as a confounding factor in the data analyses (see below).

We tested male and female adult harvest mice (age range: 57 to 743 d). We previously presented repeatability and consistency of activity, boldness and exploration in young and adult harvest mice of the same population (Schuster et al. 2017). Since young mice expressed less repeatable spatial recognition behaviour measured in a Y Maze, we now analysed only the data from adult mice. 28 individuals were added to the previous data set and we established three new behavioural tests (Novel Environment, Scare Test, and Spatial Orientation Task) to measure more traits. Behavioural tests were conducted between June 2013 and February 2015. Mice were observed between 8 am and 8 pm, and did not pass more than three different behavioural tests per day. Animals conducted the Open Field (followed by Novel Object), Scare Test and Y Maze in a randomized order. Both trials of the Novel Environment and the Spatial Orientation Task were done afterwards, also in a randomized order. Test arenas were cleaned with 70% ethanol after each test to remove any olfactory cues.

### Open Field (OF)

In the OF test, rodents are expected to spend more time next to the wall of the arena due to their predisposition to avoid open space and the risk of avian predation [1]. The OF test was shown to be a suitable setup to measure boldness (the tendency of an individual to take risks; see also [47]) in rodents (e.g., [33]). Thus, we used a modified OF test to analyse boldness and activity (the general activity level of an individual) as previously described in Schuster et al. (2017). Mice were released in the middle of a round arena (diameter 51 cm) using an opaque box for transportation. Using automated video tracking (EthoVisionXT, Version 5, Noldus), we measured the total distance moved (in cm) during 4.5 min in a round arena and the time (in s) individuals spent at the inner part of the arena (unsafe area) as parameters for activity and boldness, respectively. Tracking started when the animal reached the outer zone of the arena next to the wall for the first time (safe area, 10 cm wide). We tested 96 adult harvest mice once and 90 of them also a second time after 28 to 101 d (mean = 72.5 d).

### Novel Object (NO)

To analyse exploratory behaviour we used a NO test [12]. Exploration can be defined as an individual’s reaction to a new situation, for instance, novel objects or novel environments [47]. We conducted the NO test directly after the OF test to reduce further stress as animals were already habituated to the arena setting. We used a different novel object for each trial: A small plant pot and a glass bowl. Each object was about the same size of the mice, and animals were able to explore it from all sides and also from the top by climbing on it. The novel objects were placed into the OF by the observer, 10 cm away from the wall and at a time point when the mouse sat on the opposite side of the arena. We recorded exploratory behaviour manually for 5 min after the first contact with the novel object. We quantified the time (in s) animals spent in physical contact with the novel object. For further details see Schuster et al. (2017). We tested 69 mice once and 62 of them were tested a second time after 85 to 101 d (mean = 89.9 d).

### Novel Environment (NE)

**Fig. 1:**
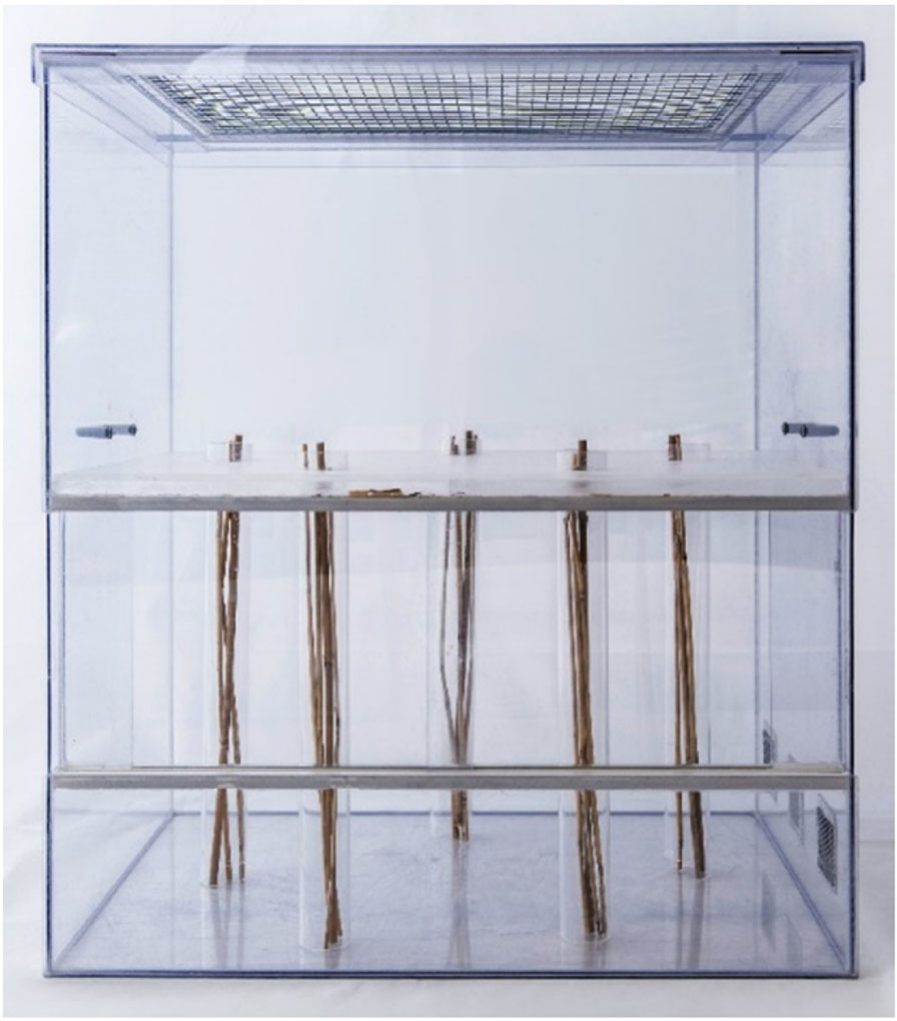
Test arena of the Novel Environment showing the five tubes connecting bottom and intermediate floor which harvest mice could explore by climbing

We established a NE test to quantify spatial exploration in harvest mice. Thereby, we adapted the idea of the NE test used by Verbeek et al. [59] analysing exploration in great tits. We here simulated a novel environment that should appeal to the study species more than the OF arena, as it offered a three-dimensional structured environment with opportunities to climb. For that, we used a polycarbonate cage (Fig. 1) similar to the home cages of the mice with a size of 58.5 cm length, 39.0 cm width and 69.5 cm height. An intermediate floor made of opaque polycarbonate was inserted at 36.0 cm height. Bottom and intermediate floor were connected by five tubes (4 cm in diameter). Each tube had an entrance (2 x 2 cm) at the bottom and was equipped with straws (replaced after each test) to ensure that mice could climb up and down through the tubes (see Fig. 1). We used two different arrangements of the tubes for the two trials. A trial lasted until animals visited all five tubes, but at least 10 min and at maximum 30 min. Mice were released at the bottom of the NE and observed directly. We defined that a mouse had visited a tube if it climbed at least one body length up or down within a tube. Because during 24 of 119 observations (20.2%) the mouse did not visit all five tubes, we used the latency (in s) until any four of the five tubes were visited by the mouse as a parameter for exploration. Eight individuals did not visit four tubes during 30 min and were scored with 1800 s. We tested 60 adult harvest mice once and 59 of them were tested a second time after 23 to 35 d (mean = 29.9 d).

### Scare Test (ST)

We used the *Scare Test* [14] to analyse the reaction towards disturbances in a familiar environment. We used the individual home cages as testing arenas and animals were observed directly. A 20 cm wide sliding door at the front side of the polycarbonate cage was regularly used during all manipulations inside the home cage. For the test, the observer fully opened the sliding door during an active phase of the mouse (e.g., the mouse was moving around in its cage), and closed it again immediately. The animals instantly hid under the hay or behind the coco coir mat in the back of the cage. We measured the latency (in s) until they returned from their hiding places as a parameter for boldness. If a mouse did not return from the hiding place within 15 min, we aborted the trial and scored the animal with 900 s (*N* = 9 tests). 56 adult harvest mice were tested once and 38 of them were tested a second time after 2 to 218 d (mean = 78.3 d).

### Y Maze (YM)

We quantified spatial recognition in an adapted YM arena [41], which relies on the assumption that rodents explore novel environments more than already known environments [34]. Known environments can be recognised through association with known object cues. In the YM arena, objects that were only visible from one arm of the arena enabled the animals to recognise whether they had previously visited a specific arm, based on spatial association. The YM test consisted of two runs. During the first run, one of the arms was locked (unknown arm), in the second run all three arms were accessible. For a more detailed description of the test see Schuster et al. (2017). We video tracked animals using EthoVisionXT software and measured the total distance moved (in cm) during the first 5 min of the second run as a parameter for activity. In animal cognition research, the behaviour of spending more time in the unknown arm of a Y Maze in the second run is commonly defined as spatial recognition in the sense that an animal recognized that there is an unknown environment (e.g., [15]). Thus, we recorded the time (in s) spent in the unknown arm during the first 5 min of the second run as a parameter for spatial recognition. We excluded one animal from further analyses as it ran straight into the known arm at the beginning of the run and did not leave this arm again. We tested 96 adult harvest mice once and 89 of them were tested a second time after 40 to 99 d (mean = 76.1 d).

### Spatial Orientation Task (SOT)

**Fig. 2:**
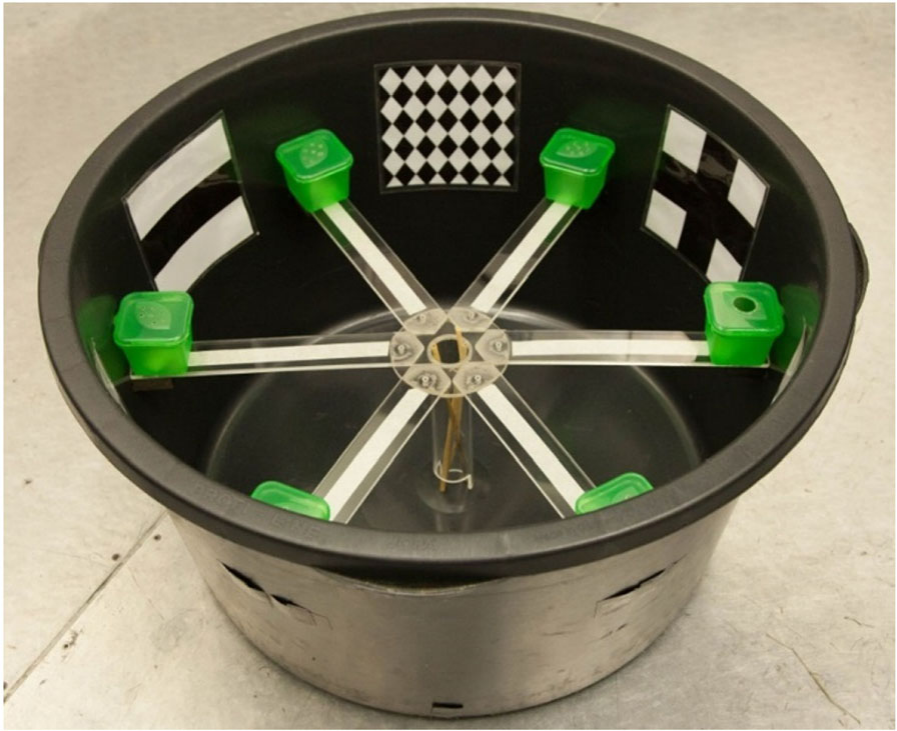
Experimental set-up of the Spatial Orientation Task. Six-arm maze elevated 20 cm above the ground with target box on the right and optical cues for spatial orientation between the arms

To evaluate individual spatial learning performance, as well as decision speed and accuracy, we established the *Spatial Orientation Task* [64]. We used a round arena, 51 cm in diameter. An elevated six arm maze was placed inside the arena, 20 cm above the bottom. Mice reached the maze by climbing through a centrally placed tube (4 cm in diameter, see Fig. 2). We placed a green plastic box (5 x 5 x 5 cm) filled with hay at the outer end of each arm. Mice could enter only the target box via a 2 cm hole in the lid. The other five boxes had closed but perforated lids, so that olfactory cues were the same for each box. As boxes were opaque, mice could not see the holes in the lid while walking on the arms of the maze. The SOT consisted of 10 or 20 learning runs (run number) conducted on two consecutive days (test day; run 1-10 on the first day, run 11-20 on the second day). Each learning run ended 1 min after the mouse had entered the target box and left again (maximum: 10 min). We did not catch the animals in close vicinity to the target box to avoid any negative associations. Mice were caught using an opaque transportation box, in which they also spent the time between learning runs. During the runs mice were supposed to learn the location of a target box (Fig. 2) which offered a hide. In the first experiments (*n* = 33) all mice conducted 20 learning runs. For all remaining tests, we stopped testing after 10 learning runs, if the mouse reached the learning criterion on that day. Optical cues attached to the wall of the arena (Fig. 2) served as landmarks that the animals could use for orientation, and for association between the position of the target box and both cues on either side of the box. The position of the target box was the same for all mice tested in the first trial, but we assigned the position of the six cues in the test arena randomly across individuals. The animals were filmed from above, while the observer recorded individual behaviour on a monitor placed out of sight of the mice. We scored spatial learning performance as the number of learning runs needed to fulfil the learning criterion. We defined the learning criterion as less than seven non-target boxes visited in total, within four consecutive learning runs. This criterion was defined during a pilot study [64]. If an individual did not reach the learning criterion, it was scored with 20 learning runs. After the learning criterion was fulfilled, all following runs (six to twenty runs per individual) were used to record decision speed and accuracy. We measured decision speed as the time (in s) between the moment when the mouse had reached the platform, and when it visited any box (all four paws on box/platform), multiplied by -1. If the animal visited the target box first, we scored an accurate decision. If any other box was visited first, we scored an inaccurate decision. The proportion of accurate first decisions within all learning runs *after* the learning criterion was fulfilled was defined as decision accuracy. During the second trial of the SOT (to estimate repeatability of spatial learning performance, decision speed and decision accuracy) we changed the set-up by changing the test room and the position of the target box within the six-arm maze. Further, we used six different optical cues than during the first trial. We tested 57 adult harvest mice once and 53 of them were tested a second time after 45 to 491 d (mean = 139.0 d).

### Data analyses

We performed all statistical analyses using *R* software, version 3.0.1 [46] and ASReml, version 4.1 [26]. We measured the effects of potential confounding factors due to the experimental setup, and due to individual characteristics of the mice, by fitting linear mixed models (LMM) using the “nlme” package [45] in *R*. We considered the following potential confounding factors caused by the experimental setup: The identity of the observer (observer), the date of the test performance (test date), and the time of the test performance (test time). Potential confounding individual factors were the sex of the mouse (sex), whether the mouse completed a specific test for the first or the second time (trial number), the body mass of the mouse measured directly before the test performance (body mass), the age in days of the mouse at the test date (age), the lab generation the mouse belonged to (our lab population consisted of seven generations), and whether the mouse was housed alone or in a pair (housing condition). We thus included the following confounding factors into each full model: The mouse identity (ID) as random factor (to account for repeated measures); sex, trial number, observer and housing condition as fixed factors; body mass, age, test date, test time and generation (quadratic function) as fixed covariates. As males and females might differ in their activity rhythms, we included the interaction sex*test time. Further, females harvest mice are larger than males [44] and this dimorphism might influence individual behaviours of sexes differently. We therefore included the interaction sex*body mass in the full model. Additionally, we included the run number and the test day (one or two) to the linear models fitting decision speed and accuracy of the SOT. We used backward stepwise reduction of the full model by excluding non-significant interactions first, followed by non-significant main effects (*p* < 0.05). We applied square root (activity in OF and YM, exploration in NO, boldness in OF and spatial recognition) or log (boldness in ST, exploration in NE and spatial learning performance) transformations to the dependent variables to ensure a normal distribution of the model residuals. For boldness in ST, exploration in NE, and spatial learning performance we multiplied the transformed values by -1 to simplify correct interpretations of the directions of correlation coefficients (i.e., larger values correspond to a faster exploration, faster learning, and bolder individuals). We present final models with the remaining confounding factors in the Additional file 1: Table S1. Confounding factors with significant effects were retained in the models for subsequent repeatability and correlation analyses.

### Repeatability

The narrow sense repeatability of personality and cognitive traits was analysed using the package “rptR” [42] in *R*. We calculated *adjusted repeatability* (R_A_) [42] by including confounding factors identified from LMMs before (see above). We estimated LMM based repeatability, including individual ID as random factor, based on 1000 bootstrapping runs and 1000 permutations. We display R_A_ values with standard errors and asymptotic 95% confidence intervals (CIs), and permutation based *p* values.

### Correlations between traits

We fitted multivariate LMMs using restricted maximum likelihood (REML) in ASReml4 [26] to estimate phenotypic variances for each personality and cognitive trait, as well as the phenotypic covariances between those. We partitioned the within-individual variances and covariances from the between-individual variances and covariances as recommended by Dingemanse and Dochtermann [18]. We then used these variances and covariances to calculate phenotypic correlations between the traits within individuals (r_W_) and between individuals (r_B_). We included all previously defined significant confounding factors in the fixed models. The animal ID was included as random factor to all models.

We fitted the first multivariate model for the six personality traits to test for a behavioural syndrome. We used a stepwise approach to build up the final model. This was done to ensure convergence of the complex multivariate model and to reduce the possibility that the model converges on a local likelihood optimum. First, we applied a diagonal variance model, which estimated only variances, but no covariances. Then, we applied an unstructured variance model where all between-individual and within-individual covariances were first fixed to zero. We then estimated the between-individual and within-individual covariances one by one, using estimates from respective bivariate models as starting values. We present correlations based on the final six-trait model with a fully unstructured between-individual variance-covariance matrix. The within-individual covariance matrix was modelled between activity in the OF test, activity in the YM, boldness in the OF test, and exploration in the NO test. For boldness in the ST and exploration in the NE, we were not able to model within-individual covariances, as those behaviours were measured at different time points. Thus, our modelling approach is a combination of scenario 3 and 4 described in Table 2 by Dingemanse and Dochtermann [18].

We extended the six-trait model by adding spatial recognition and spatial learning performance to test for a relationship between personality and spatial cognitive traits. We also calculated correlations based on the final eight-trait model with a fully unstructured between-individual variance-covariance matrix. Within-individual covariances with spatial learning performance in the SOT could not be estimated, as this behaviour was measured at a different time point.

Finally, we extended the six-trait model by adding decision speed and decision accuracy to test for a speed-accuracy trade-off. We again calculated correlations based on the final eight-trait model with a fully unstructured between-individual variance-covariance matrix. The within-individual covariance matrix was modelled between activity in the OF test, activity in the YM, boldness in the OF test, and exploration in the NO test. From these eight-trait models we only display between-individual correlation coefficients with *p* < 0.1, the full information is given in the supplement.

In the above described analyses, our measure for decision accuracy summarized accuracy over all learning runs after the learning criterion was fulfilled, because we were not able to conduct multivariate generalized LMMs. However, since ASReml4 allows bivariate models with one binominal response variate, we conducted additional tests for the speed-accuracy trade-off. In these analyses, decision accuracy was scored as a binary response (correct decision or not) for each learning run and was modelled with a logit link. For decision speed and accuracy, we calculated between- and within-individual correlations from a fully unstructured between- and within-individual variance-covariance matrix. For learning performance and accuracy, only the between-individual correlation could be estimated. Potential confounding fixed factors (as described above) were identified before by fitting a generalized LMM using the “lme4” package [2] in *R*. The trial number (estimate ± SE = -0.242 ± 0.123, *p* = 0.049) and the test day (-0.282 ± 0.124, *p* = 0.023) significantly influenced the decision accuracy and remained in the generalized LMM.

## Results

### Repeatability

All personality traits were significantly repeatable (Table 1). R_A_ values ranged between 0.221 and 0.598, whereby activity was the behaviour with the highest repeatability. Most cognitive traits were also significantly repeatable: Spatial recognition, spatial learning performance, and decision speed showed repeatabilities between 0.127 and 0.263 (Table 1). However, decision accuracy was not significantly repeatable. The results of the model reductions can be found in the Additional file 1: Table S1. Significant confounding factors were retained in the fixed model for all further analyses and are listed in Table 1.

**Table 1:** Repeatability (R_A_) with 95% confidence interval (CI) for each personality and cognitive trait

Personality/cognitive trait	Behavioural test	Confounding factors	N	R_A_	95% CI	*p* value
Activity	Open Field	generation + generation^2^	186/96	**0.404 ± 0.086**	[0.226, 0.552]	<0.001
	Y Maze	-	184/96	**0.598 ± 0.064**	[0.456, 0.705]	<0.001
Boldness	Scare Test	-	94/56	**0.256 ± 0.145**	[0.000, 0.534]	<0.001
	Open Field	trial number + observer	186/96	**0.319 ± 0.097**	[0.115, 0.502]	<0.001
Exploration	Novel Object	housing condition	131/69	**0.328 ± 0.113**	[0.097, 0.531]	<0.001
	Novel Environment	trial number	119/60	**0.221 ± 0.119**	[0.000, 0.432]	<0.001
Spatial recognition	Y Maze	-	184/96	**0.200 ± 0.104**	[0.000, 0.429]	0.002
Spatial learning performance	Spatial Orientation Task	trial number	110/57	**0.127 ± 0.116**	[0.000, 0.382]	0.042
Decision accuracy	Spatial Orientation Task	-	102/56	0.125 ± 0.122	[0.000, 0.411]	1.000
Decision speed	Spatial Orientation Task	observer + run number	1156/56	**0.263 ± 0.027**	[0.211, 0.313]	<0.001

Generation was fitted as a quadratic function. Sample sizes (N) are given as observations/individuals. Significant repeatability values are marked bold

In the Spatial Orientation Task all 57 harvest mice were able to learn the position of the target box, at least in one of the two trials. Six individuals did not reach the learning criterion within the 20 learning runs, three during the first trial and three during the second trial. 53.6% of the animals reached the learning criterion already within the first five runs. We provide some examples of observed learning curves in the supplement (see Additional file 2).

### Correlations between traits

**Fig. 3:**
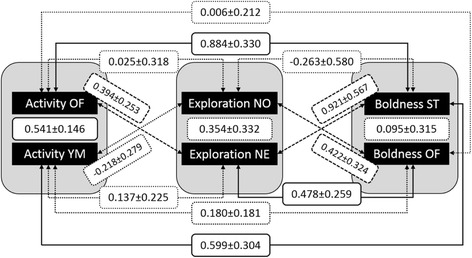
Behavioural syndrome: Between-individual correlations (± standard errors) between personality traits calculated from a multivariate mixed model. Solid lines indicate significant between-individual correlations (*p* < 0.05), dashed lines indicate tendencies (*p* < 0.1), and dotted lines indicate non-significant between-individual correlations. OF: Open Field, YM: Y Maze, NO: Novel Object, NE: Novel Environment, ST: Scare Test

We observed four significant positive between-individual correlations among personality traits, three more relationships showed a tendency (*p* < 0.1) with high positive between-individual correlation coefficients (Fig. 3). The two measures of activity showed a significant positive between-individual correlation (r_B_ = 0.541 ± 0.146, *p* < 0.001). Both activity measures correlated significantly with boldness measured in the Scare Test, but not with boldness measured in the Open Field test. However, exploration in the Novel Environment correlated significantly with boldness in the Open Field test. There were two further relationships between boldness and exploration, which tended to correlate positively (see Fig. 3). Finally, exploration in the Novel Environment tended to correlate positively with activity in the Open Field test. The two measures of exploration showed no significant between-individual relationship, nor did the two measures of boldness.

We observed only one significant within-individual correlation between the activity in the Open Field test and activity in the Y Maze (r_W_ = 0.257 ± 0.108, *p* = 0.009). All other modelled within-individual correlation coefficients were very small and not significant (see Table 2).

**Table 2:** Behavioural Syndrome: Within-individual correlations between personality traits calculated from a multivariate mixed model

	Activity OF	Activity YM	Boldness OF	Boldness ST	Exploration NO	Exploration NE
Activity OF		**0.257 ± 0.108**	-0.006 ± 0.106	NA	0.082 ± 0.126	NA
Activity YM	0.009		-0.040 ± 0.116	NA	0.065 ± 0.125	NA
Boldness OF	0.477	0.364		NA	0.062 ± 0.125	NA
Boldness ST	NA	NA	NA		NA	NA
Exploration NO	0.256	0.301	0.311	NA		NA
Exploration NE	NA	NA	NA	NA	NA	

Within-individual correlation coefficients (± standard errors) are shown above, and *p* values below the diagonal

Significant correlation is marked in bold. NA: within-individual correlation not modelled due to missing data. *OF* Open Field, *YM* Y Maze, *NO* Novel Object, *NE* Novel Environment, *ST* Scare Test

**Fig. 4:**
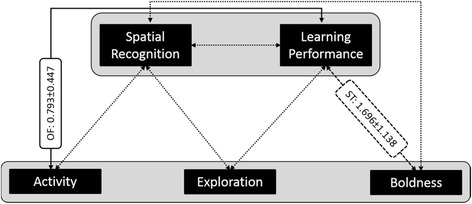
Relationship between personality and spatial cognition: Between-individual correlations (± standard errors) between personality traits and cognitive traits calculated from an eight-trait multivariate mixed model, but only five traits are displayed (see explanation in the text). The solid line indicates the significant correlation (*p* < 0.05) between activity in the Open Field (OF) and learning performance in the Spatial Orientation Task, the dashed line indicates the tendency (*p* < 0.1) between boldness in the Scare Test (ST) and learning performance in the Spatial Orientation Task, and dotted lines indicate non-significant correlations between the other traits

When we extended this six-trait model by spatial recognition and spatial learning performance, our conclusions from the between-individual and within-individual correlations among personality traits did not change (see Additional file 1: Tables S1 and S3). In the eight-trait model, we identified a strong and significant between-individual correlation among activity in the Open Field and spatial learning performance (r_B_ = 0.793, see Fig. 4) and a tendency for a strong and positive between-individual correlation among spatial learning performance and boldness in the Scare Test (r_B_ = 1.696, see Fig. 4). Further non-significant between-individual and within-individual correlation coefficients are not displayed in Fig. 4, but all results of the multivariate mixed model can be found in the Additional file 1: Tables S2 and S3. There was no significant between-individual correlation, nor any within-individual correlation between spatial recognition and any personality trait. The between-individual correlation coefficient between spatial learning performance and spatial recognition was large and positive (r_B_ = 0.562 ± 0.594), but not significant (*p* = 0.172).

**Fig. 5:**
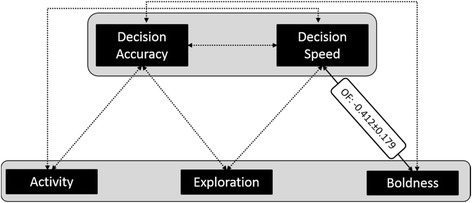
Test for a relationship between personality traits and decision speed and accuracy based on a speed-accuracy trade-off: Between-individual correlations (± standard errors) between personality traits, and decision speed and accuracy calculated from an eight-trait multivariate mixed model, but only five traits are displayed (see explanation in the text). The solid line indicates the significant correlation (*p* < 0.05) between activity in the Open Field (OF) and decision speed in the Spatial Orientation Task, dotted lines indicate non-significant correlations between the other traits

When we extended the six-trait model by decision speed and decision accuracy, between-individual and within-individual correlations among personality traits did also not change meaningfully (see Additional file 1: Tables S4 and S5). In this eight-trait model, we identified a significant negative between-individual correlation between boldness in the Open Field and decision speed (r_B_ = -0.412 ± 0.179, see Fig. 5). Further non-significant between-individual and within-individual correlation coefficients are not displayed in Fig. 5, but all results of the multivariate mixed model can be found in the Additional file 1: Tables S4 and S5. There was no significant between-individual correlation among decision speed and decision accuracy (r_B_ = 0.207 ± 0.425, *p* = 0.313).

The bivariate generalized LMM with decision accuracy as binary response and decision speed with normal distribution resulted in a within-individual correlation coefficient of r_W_ = 0.023 ± 0.020 (*p* = 0.125) and a between-individual correlation coefficient of r_B_ = 0.018 ± 0.248 (*p* = 0.471). The model with decision accuracy as binary response and learning performance did not converge due to the very low between-individual variance for accuracy. If we fixed that variance to 0.11 (this was 2% of the observed within-individual variance for this trait, and the smallest value that allowed model convergence), the between-individual correlation coefficient was r_B_ = 0.246 ± 0.261 (*p* = 0.347). Any larger estimate of between-individual variance for accuracy produced smaller (and never significant) correlation coefficients.

## Discussion

We found strong evidence for a behavioural syndrome in Eurasian harvest mice. As we expected, activity, boldness and exploration showed positive correlations and formed a fast-slow-behavioural syndrome [56]. Further, we found some indication for a relationship between spatial learning performance and personality types. More active harvest mice reached the learning criterion in the Spatial Orientation Task earlier than less active individuals. The speed-accuracy trade-off was not apparent within, nor between individuals. Nevertheless, we found weak evidence for a relationship between personality and cognitive traits as shyer harvest mice made decisions faster than bolder mice. Thus, our data partly support the hypothesis that behavioural types may correlate with individual cognitive ability [53].

### Repeatability

One basic assumption of animal personality is the repeatability of behaviours [47]. We here confirmed this assumption for all personality traits tested in adult Eurasian harvest mice. R_A_ values were between 0.221 and 0.598, which lies within the range of usually observed repeatabilities of personality traits [4, 25]. We previously reported similar repeatabilities for a smaller dataset on juvenile and adult harvest mice from the same laboratory population [48].

Spatial recognition and spatial learning performance were also significantly repeatable in adult harvest mice. Lantová et al. [38] already observed that common voles (*Microtus arvalis*) performed repeatably in an eight-arm radial arm maze. Voles thereby showed repeatable maze-exploring tactics and also the maze exploration activity was significantly repeatable [38]. Currently, there is only scarce evidence for repeatability of spatial recognition and general learning ability in animals [27]. In black-capped chickadees (*Poecile atricapillus*), the number of trials to reach a learning criterion in a colour association task was repeatable [31]. Further, Carib grackles *(Quiscalus lugubris*) behaved consistently in three different types of learning tasks and solved these tasks consistently well [20]. Repeatability and consistency of cognitive traits results in less flexible behaviour of individuals in situations when cognitive abilities are needed. This reduced flexibility may cause a constraint to the expression of adaptive behaviour. It should be noted, however, that studies on within-species variation in cognitive abilities (including our study) generally focus on few test situations for specific cognitive tasks. Individuals with low performance in those tasks may show stronger cognitive abilities in other tasks. It remains to be shown, which trade-offs cause repeatable reduced cognitive ability in a specific test.

Among the two traits that characterise decision styles, decision speed was repeatable in harvest mice, whereas decision accuracy was not. The only other published study on repeatability of decision speed and accuracy was conducted on zebrafish (*Danio rerio*), where Wang et al. [60] showed that both, speed and accuracy, were significantly repeatable in a spatial colour discrimination task. Harvest mice may indeed not behave as repeatably during decisions as zebrafish did, or we may have failed to measure decision accuracy adequately. Our test design was based on the assumption that the mice have a high motivation to inspect the target box (their hide in the test arena) as soon as they were released into the arena. However, after a few runs, some mice may not have been motivated any more to visit the hide, but rather preferred to explore the remainder of the test arena. As there is no other evidence available so far, more experimental work is needed to better understand the repeatability of decision accuracy and speed in animals in general.

### Behavioural syndrome

We found significant positive between-individual correlations or positive tendencies between the three studied personality traits. Activity, exploration and boldness thus form a *fast-slow-behavioural syndrome* [56] in our study species. This is in line with a large body of personality literature in rodents: Starting with the observations in laboratory house mice [36], more and more studies reported fast-slow-behavioural syndromes (e.g., in Belding’s ground squirrels, *Urocitellus beldingi*; [19], and in cavies, *Cavia aperea*; [28]). However, some behavioural correlations were not stable over ontogeny (e.g., [29, 35]), and in some species, no behavioural syndrome was detected (e.g., in yellow-bellied marmots, *Marmota flaviventris;* [43]).

In our study, a number of tested relationships between personality traits showed reasonable between-individual correlation coefficients, but large standard errors. This suggests that our current sample size (56 to 96 individuals per trait) might be too small to fully describe the relationship between all measured behaviours. Alternatively, within-individual covariance might, if not estimated, mask or inflate between-individual covariance. Only between-individual covariances are indicative for a behavioural syndrome [18]. Since we did not conduct all behavioural tests at the same time, we were not able to model all within-individual covariances. However, among those relationships, where we did estimate within-individual covariance, it was significant only between the two measures of activity. In that case, an alternative model excluding within-individual covariances did not lead to different conclusions about the relationships between the traits (data not shown). We thus conclude that, in our test situation, within-individual covariances did not largely influence phenotypic correlations, and that higher sample sizes might allow to estimate correlations with higher accuracy. Further, the lack of significant correlations between the two measures of explorative behaviour (Novel Object and Novel Environment), and between the two measures of boldness (Scare Test and Open Field) may indicate a suboptimal behavioural test selection. These tests may indeed measure different behaviours. Open Field and Novel Object tests are frequently used to measure either exploration, activity, boldness or neophobia. Different experimenters thereby apply different methods to quantify the measured behaviours (e.g., boldness as the latency to reach novel objects; [28], or boldness as the latency to enter the middle of an Open Field; [33]). However, since behavioural syndromes assume relationships between similar behavioural traits in different situations, we would still expect correlations between slightly different measures of boldness and exploration. We cannot exclude that larger sample sizes may reveal correlations between these measures. Currently, less individuals were tested in the Novel Environment and in the Scare Test, compared to the Novel Object and the Open Field, respectively.

We detected positive correlations between activity, exploration and boldness. These relationships could arise from constraints in proximate mechanisms, such as physiological pathways, maintaining these behaviours [51]. Alternatively, selection for alternative types may maintain fast and slow behavioural types in a population. This type of disruptive selection can be caused by variation in selective agents in the environment (e.g., fluctuation of food availability in great tits, *Parus major*; [17]), or by alternative behavioural optima during different life-history stages (e.g., life-history trade-off between current and future reproduction in grey mouse lemurs, *Microcebus murinus*; [13]). Harvest mice live in a very variable environment where it is likely that fitness advantages for different behavioural types change during the year. Like in other rodent species, population sizes of harvest mice increase dramatically during the summer and show high peaks in autumn, followed by a marked reduction in population size over winter [44]. More active, bolder and more explorative individuals may have higher fitness in the more competitive situations during high population density. Then, the fast behavioural type may gain better access to suitable nest sites, food and mating partners. Slow (reactive) harvest mice on the other hand may have fitness advantages during winter and spring, when population density is lower. During this time, the slow behavioural type may save energy through lower levels of activity and exploration, and potentially fewer interactions with conspecifics. However, selection for behavioural types will only result in the maintenance of these types in the population if the correlations that underlie the behavioural syndrome are in fact genetic relationships. We here present phenotypic trait correlations, and it remains to be tested if these relationships are also genetically based.

### Relationship between personality and cognitive traits

As expected, we found some indication for a relationship between personality types and spatial learning performance. Harvest mice that were more active in the Open Field reached the learning criterion in the Spatial Orientation Task earlier than less active individuals. Further, a non-significant tendency suggested a positive relationship between boldness in the Scare Test and spatial learning performance. Finally, the between-individual correlation coefficient between spatial learning performance and boldness in the Open Field test showed a large correlation coefficient, with large standard error (Additional file 1: Table S2). Taken together, we found some weak indication that mice of different personality types may differ in their performance to learn. Some recent studies have suggested such a relationship: Fast behavioural types demonstrated faster conditioning learning in Panamanian bishop fish (*Brachyrhaphis episcopi*), faster association learning in cavies (*Cavia aperea*; [16, 28]), and steeper generalization gradients in pigeons (*Columba livia;* [30]). The difference in (spatial) learning performance is assumed to result from differences in how individuals assess and attend to the learning situation [53]. However, no such relationship was reported in Eastern water skinks (*Eulamprus quoyii*) and in common carp (*Cyprinus carpio*), where bold as well as shy animals learned spatial association tasks equally successful [9, 40]. And also in black-capped chickadees (*Poecile atricapillus*), learning speed did not predict exploratory behaviour [31].

We expected that mice which learned the orientation task faster, would also show better spatial recognition. In the Y Maze, we measured spatial recognition as the association between unknown landmarks and a new environment, and in the Spatial Orientation Task, the position or the landmarks of a specific environment had to be memorized. Since both task designs offered visual cues (landmarks) for spatial recognition, mice may have employed similar cognitive pathways in both tests. Although the between-individual correlation coefficient between spatial recognition and learning performance was large and positive, it did not reach significance due to the large standard error (Additional file 1: Table S2). This large error variance may occur because individuals differed in their motivation to solve the orientation task. If some mice were indeed more interested to explore the six arm maze rather than to inspect the target box, we have to assume a large error in our measure of learning performance. The challenge remains to design a spatial learning task that ensures high task solving motivation in all individuals without restricting the test situation to a feeding context.

We did not observe any of the expected correlations between personality traits and spatial recognition in the Y Maze. The time animals spent in the unknown arm of the Y Maze was not related to activity, exploration or boldness of the individuals, as measured in other behavioural tests. Mamuneas et al. [39] could also not identify any differences in the spatial recognition between shy and bold three-spined sticklebacks (*Gasterosteus aculeatus*) tested in a T Maze where landmarks could be associated with food rewards in one of the arms. Even if behavioural types do not differ in their spatial recognition, they may still use different, equally successful strategies to recognize known areas or to build associations with specific optical cues. In our test for spatial recognition, mice could recognize the unknown arm based on landmarks that we placed around the arms. Thereby, the animals’ view was blocked to the side of the Y Maze, but they could have used cues above them (e.g., the camera, or structures in the ceiling of the room) to define a known position in the room. However, mice may also have recognized the unknown arm based on *path integration* – by keeping track of their own location in relation to a known position in their environment [49]. We were not able to test if mice of different personality types employed different orientation strategies. There is indeed evidence for this in common carp (*Cyprinus carpio*) where different personality types solved an orientation task using different types of information to get access to a food reward [40]. Reactive (slow) individuals followed a light cue that was associated with food, while proactive (fast) animals formed fixed movement routines to reach the location of the food reward [40].

Although the repeatability of decision speed suggested the presence of decision styles in harvest mice, they did not face a speed-accuracy trade-off in the Spatial Orientation Task: Individuals that decided more quickly did not make more or less accurate decisions than slow deciding individuals. Also, fast learning individuals did not make more or less accurate decisions once they had learned the task. However, given our concerns about the motivation of the mice to seek shelter in the Spatial Orientation Task (see above), it appears premature to draw strong conclusions from this observation. We will need to design more specific tests for decision speed and accuracy, to measure the correlation between them, also in further contexts. Both, decision speed and accuracy did not correlate significantly with any personality trait, with one exception: Shy harvest mice, which spent less time in the unsafe (middle) part of the Open Field decided faster in the Spatial Orientation Task. This finding is in contrast to the theoretical expectation that slow (and shy) behavioural types make slow, but accurate decisions [53]. In our specific setup, shy mice (that spent less time in the middle of the Open Field) might have preferred to leave the exposed position in the middle of the Spatial Orientation Task arena faster, than bold individuals, and thus expressed a faster decision behaviour in that test. Under a speed-accuracy trade-off, this behaviour could indeed lead to a lower spatial learning performance due to more frequent inaccurate decisions. Our data did not exclude the possibility that less active individuals may actually have decided less accurately: The two relevant correlation coefficients between decision accuracy and activity were large and positive, albeit not significant due to high variation in the data (reflected in large standard errors, see Additional file 1: Table S4). However, overall, we found little support for the hypothesis that harvest mice show a relationship between behavioural types and decision making behaviour.

## Conclusions

Our results showed that adult Eurasian harvest mice behaved repeatably and that they expressed a behavioural syndrome with strong positive correlations between the three personality traits activity, exploration, and boldness. Further, we showed that more active and bolder individuals (the fast behavioural type) were faster in learning the Spatial Orientation Task than mice expressing the slow behavioural type. We thus found support for Sih and Del Giudice’s [53] hypothesis that there is a relationship between personality and cognitive traits. If fast behavioural types in general learn more quickly than slow behavioural types, we should wonder why both variants still persist in animal populations. First, slow learners may gain advantages in variable environments as they may be better able to adapt or reverse learned behaviour (e.g., [32]). Second, shy and less active harvest mice may indeed be the more adapted behavioural type during some parts of the year, when predation risk is particularly high (e.g., during times of reduced vegetation cover). Thus, the observed relationship may document non-independent trait evolution that maintains slow learners in the population. We found only weak indications for a relationship between personality and decision making behaviour. This suggests that decision styles and behavioural types can evolve independently from each other in this species. However, as current results (including this study) are contradictory, more investigations are needed to better understand the relationship between cognitive performance, decision styles and personality in animals.

## Additional files

Additional file 1: Table S1. Final LMMs with significant confounding factors on personality and cognitive traits in harvest mice. **Table S2.** Between-individual correlations among personality and cognitive traits (spatial recognition and spatial learning performance) in harvest mice, calculated from a multivariate mixed model. **Table S3.** Within-individual correlations between personality and cognitive traits (spatial recognition and spatial learning performance) in harvest mice, calculated from a multivariate mixed model. **Table S4.** Test for a cognitive syndrome caused by a speed-accuracy trade-off in harvest mice: Between-individual correlations among personality traits and decision styles (decision speed and accuracy) calculated from a multivariate mixed model. **Table S5.** Test for a cognitive syndrome caused by a speed-accuracy trade-off in harvest mice: Within-individual correlations between personality traits and decision styles (decision speed and accuracy) calculated from a multivariate mixed model. (PDF 324 kb)

Additional file 2: Examples of learning curves for individual mice tested in the Spatial Orientation Task. (PDF476 kb)
