# Supplementary material for: A behavioural syndrome, but less evidence for a relationship with cognitive traits in a spatial orientation context
Source: Front Zool. 2017 Mar 24;14:19. doi: 10.1186/s12983-017-0204-2 (PMC5364594; doi:10.1186/s12983-017-0204-2)
Supplement: Supplementary file 1 — Final LMMs with significant confounding factors on personality and cognitive traits in harvest mice. Table S2. Between-individual correlations among personality and cognitive traits (spatial recognition and spatial learning performance) in harvest mice, calculated from a multivariate mixed model. Table S3. Within-individual correlations between personality and cognitive traits (spatial recognition and spatial learning performance) in harvest mice, calculated from a multivariate mixed model. Table S4. Test for a cognitive syndrome caused by a speed-accuracy trade-off in harvest mice: Between-individual correlations among personality traits and decision styles (decision speed and accuracy) calculated from a multivariate mixed model. Table S5. Test for a cognitive syndrome caused by a speed-accuracy trade-off in harvest mice: Within-individual correlations between personality traits and decision styles (decision speed and accuracy) calculated from a multivariate mixed model. (PDF 324 kb) [file 12983_2017_204_MOESM1_ESM.pdf]

Supplementary Table 1 Final LMMs with significant confounding factors on personality and cognitive traits in harvest mice. Sample sizes (N) are given as observations/individuals. Generation was fitted as a quadratic function. SE = standard error.

personality/cognitive trait	behavioural test	N	confounding factors	estimate ± SE	p value
**activity**	Open Field	186/96	generation	- 0.848±0.247	< 0.001
			generation²	+0.118±0.032	< 0.001
	Y Maze	184/96	-		
**boldness**	Scare Test	94/56	-		
	Open Field	186/96	trial number	+0.327±0.117	0.006
			observer	- 0.377±0.149	0.013
**exploration**	Novel Object	131/69	housing condition	+0.544±0.182	0.004
	Novel Environment	119/60	trial number	+0.584±0.148	< 0.001
**spatial recognition**	Y Maze	184/96	-		
**spatial learning ability**	Spatial Orientation Task	110/57	trial number	- 0.378±0.175	0.035
**decision accuracy**	Spatial Orientation Task	102/56	-		
**decision speed**	Spatial Orientation Task	1156/56	observer	- 0.117±0.015	< 0.001
			run number	+0.025±0.005	< 0.001

We observed that the trial number had a significant effect on three variables. Boldness increased in the second trial of the Open Field, and exploration also increased in the second trial of the Novel Environment as mice visited four tubes faster than in the first trial. These effects were likely caused by a habituation of the mice to the test arenas. Spatial learning ability decreased in the second trial (mice reached the learning criterion on average later than during the first trial). Here, mice might have been less motivated to reach the target box during the second trial, as they recognized the general test setup. With increasing run numbers during the Spatial Orientation Task, mice decided faster, which may indicate learning and memory of the target box position. The observer had a significant effect on boldness in the Open Field and on decision speed in the Spatial Orientation Task, which could be due to differences in handling of the animals. Harvest mice housed in pairs were further more explorative in the Novel Object test. We discussed confounding factors in more detail in Schuster et al. (submitted).

Supplementary Table 2 Between-individual correlations among personality and cognitive traits (spatial recognition and spatial learning ability) in harvest mice, calculated from a multivariate mixed model. Between-individual correlation coefficients (± standard errors) are shown above, and p values below the diagonal. Significant correlations are marked in bold, tendencies are printed in italics. OF = Open Field, YM = Y Maze, NO = Novel Object, NE = Novel Environment, ST = Scare Test.

	**activity OF**	**activity YM**	**boldness OF**	**boldness ST**	**exploration NO**	**exploration NE**	**spatial recognition**	**learning ability**
**activity OF**		**0.514±0.149**	-0.019±0.212	**0.884±0.400**	0.028±0.319	*0.371±0.257*	0.024±0.313	**0.793±0.447**
**activity YM**	0.000		0.175±0.182	**0.590±0.353**	-0.230±0.280	0.148±0.227	-0.066±0.279	-0.216±0.326
**boldness OF**	0.465	0.168		0.201±0.345	*0.437±0.325*	**0.453±0.263**	-0.102±0.322	0.508±0.407
**boldness ST**	0.014	0.047	0.280		-0.209±0.619	*1.106±0.689*	0.208±0.504	*1.696±1.138*
**exploration NO**	0.465	0.206	0.089	0.368		0.359±0.335	-0.183±0.526	0.265±0.475
**exploration NE**	0.074	0.257	0.042	0.054	0.142		0.280±0.394	-0.030±0.412
**spatial recognition**	0.470	0.407	0.376	0.340	0.364	0.239		0.562±0.594
**learning ability**	0.038	0.254	0.106	0.068	0.288	0.471	0.172	

Supplementary Table 3 Within-individual correlations between personality and cognitive traits (spatial recognition and spatial learning ability) in harvest mice, calculated from a multivariate mixed model. Within-individual correlation coefficients (± standard errors) are shown above, and p values below the diagonal. The significant correlation is marked in bold. NA: within-individual correlation not modelled due to missing data. OF = Open Field, YM = Y Maze, NO = Novel Object, NE = Novel Environment, ST = Scare Test.

	**activity OF**	**activity YM**	**boldness OF**	**boldness ST**	**exploration NO**	**exploration NE**	**spatial recognition**	**learning ability**
**activity OF**		**0.264±0.107**	-0.007±0.106	NA	0.084±0.126	NA	0.092±0.109	NA
**activity YM**	0.007		-0.045±0.116	NA	0.073±0.125	NA	0.041±0.106	NA
**boldness OF**	0.474	0.348		NA	0.059±0.125	NA	-0.086±0.111	NA
**boldness ST**	NA	NA	NA		NA	NA	NA	NA
**exploration NO**	0.251	0.278	0.319	NA		NA	0.094±0.119	NA
**exploration NE**	NA	NA	NA	NA	NA		NA	NA
**spatial recognition**	0.198	0.348	0.218	NA	0.215	NA		NA
**learning ability**	NA	NA	NA	NA	NA	NA	NA	

Supplementary Table 4 Test for a cognitive syndrome caused by a speed-accuracy trade-off in harvest mice: Between-individual correlations among personality traits and decision styles (decision speed and accuracy) calculated from a multivariate mixed model. Between-individual correlation coefficients (± standard errors) are shown above, and p values below the diagonal. Significant correlations are marked in bold, tendencies are printed in italics. OF = Open Field, YM = Y Maze, NO = Novel Object, NE = Novel Environment, ST = Scare Test.

	activity OF	activity YM	boldness OF	boldness ST	exploration NO	exploration NE	decision speed	decision accuracy
**activity OF**		**0.546±0.145**	0.016±0.212	**0.877±0.351**	0.023±0.316	*0.408±0.254*	0.179±0.190	0.606±0.672
**activity YM**	0.000		0.177±0.182	**0.593±0.318**	-0.220±0.278	0.140±0.227	0.164±0.173	1.136±0.999
**boldness OF**	0.470	0.166		0.159±0.325	0.412±0.324	**0.469±0.262**	**-0.412±0.179**	0.166±0.517
**boldness ST**	0.006	0.031	0.312		-0.402±0.592	*0.928±0.593*	-0.393±0.385	0.190±1.167
**exploration NO**	0.472	0.214	0.102	0.248		0.342±0.333	0.093±0.243	-0.357±0.680
**exploration NE**	0.054	0.270	0.037	0.059	0.152		-0.134±0.222	-0.079±0.557
**decision speed**	0.173	0.172	0.011	0.154	0.351	0.274		0.207±0.425
**decision accuracy**	0.184	0.128	0.374	0.435	0.300	0.443	0.313	

Supplementary Table 5 Test for a cognitive syndrome caused by a speed-accuracy trade-off in harvest mice: Within-individual correlations between personality traits and decision styles (decision speed and accuracy) calculated from a multivariate mixed model. Within-individual correlation coefficients (± standard errors) are shown above, and p values below the diagonal. The significant correlation is marked in bold. NA: within-individual correlation not modelled due to missing data. OF = Open Field, YM = Y Maze, NO = Novel Object, NE = Novel Environment, ST = Scare Test.

	activity OF	activity YM	boldness OF	boldness ST	exploration NO	exploration NE	decision speed	decision accuracy
**activity OF**		**0.258±0.108**	-0.011±0.106	NA	0.067±0.125	NA	NA	NA
**activity YM**	0.008		-0.041±0.116	NA	0.083±0.126	NA	NA	NA
**boldness OF**	0.461	0.364		NA	0.061±0.125	NA	NA	NA
**boldness ST**	NA	NA	NA		NA	NA	NA	NA
**exploration NO**	0.296	0.255	0.313	NA		NA	NA	NA
**exploration NE**	NA	NA	NA	NA	NA		NA	NA
**decision speed**	NA	NA	NA	NA	NA	NA		NA
**decision accuracy**	NA	NA	NA	NA	NA	NA	NA	
