# Supplementary material for: A behavioural syndrome, but less evidence for a relationship with cognitive traits in a spatial orientation context
Source: Front Zool. 2017 Mar 24;14:19. doi: 10.1186/s12983-017-0204-2 (PMC5364594; doi:10.1186/s12983-017-0204-2)

Supplementary Material

Examples of learning curves for individual mice tested in the Spatial Orientation Task.

The mice had to learn the location of a target box (out of six boxes, see Figure 2) which offered a hide. The learning criterion was reached when less than seven non-target boxes were visited in total, within four consecutive learning runs.

Each line represents a learning curve of an individual mouse. Quick learners reached the criterion on the first day (between learning run 1 to 10), see plot (A). Slow learners reached the criterion on the second day (between learning run 11 to 20), see plot (B). Some mice did not reach the learning criterion (never learned), see plot (C). Note that mouse 169 was a slow learner in the first trial, but never reached criterion in the second trial.

(A)

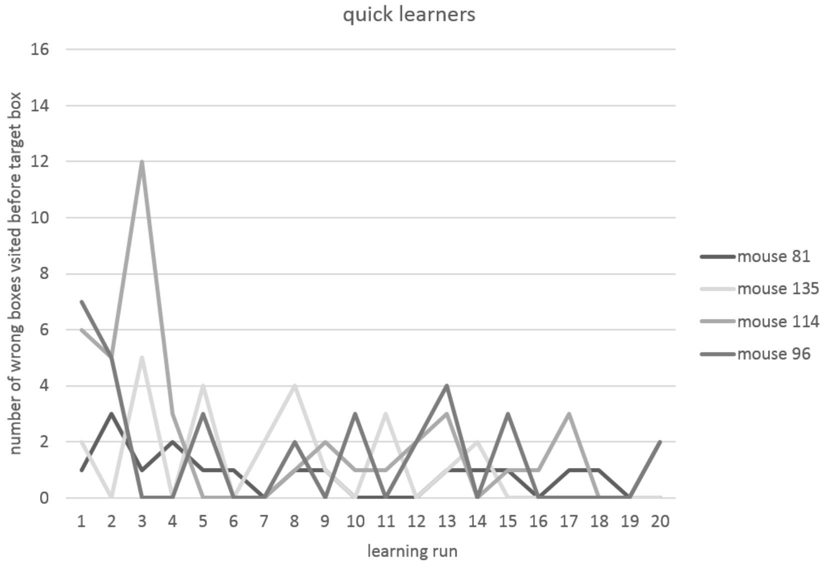

(B)

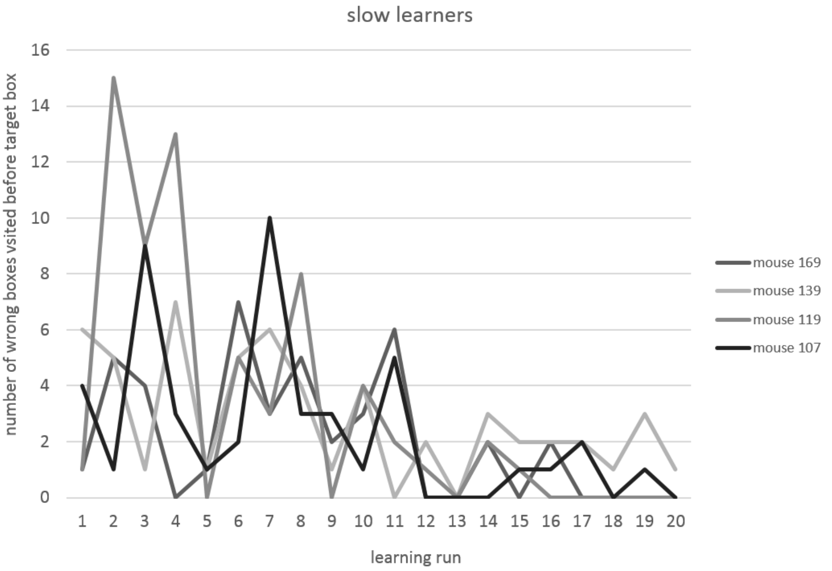

(C)

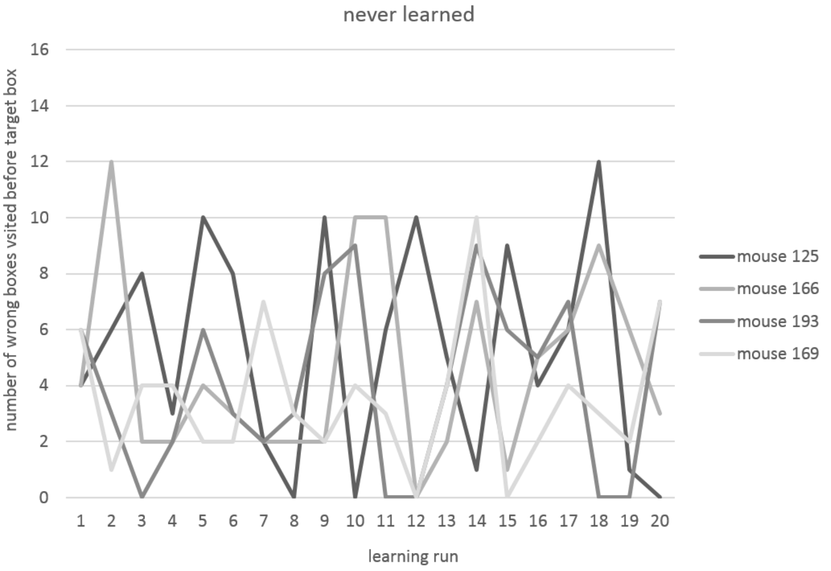

Supplement: Supplementary file 2 — Examples of learning curves for individual mice tested in the Spatial Orientation Task. (PDF476 kb) [file 12983_2017_204_MOESM2_ESM.pdf]
